# Astrocytes and Developmental Plasticity in Fragile X

**DOI:** 10.1155/2012/197491

**Published:** 2012-07-11

**Authors:** Connie Cheng, Mary Sourial, Laurie C. Doering

**Affiliations:** Department of Pathology and Molecular Medicine, McMaster University, HSC 1R1, 1280 Main Street West, Hamilton, ON, Canada L8S 4K1

## Abstract

A growing body of research indicates a pivotal role for astrocytes at the developing synapse. In particular, astrocytes are dynamically involved in governing synapse structure, function, and plasticity. In the postnatal brain, their appearance at synapses coincides with periods of developmental plasticity when neural circuits are refined and established. Alterations in the partnership between astrocytes and neurons have now emerged as important mechanisms that underlie neuropathology. With overall synaptic function standing as a prominent link to the expression of the disease phenotype in a number of neurodevelopmental disorders and knowing that astrocytes influence synapse development and function, this paper highlights the current knowledge of astrocyte biology with a focus on their involvement in fragile X syndrome.

## 1. Introduction

In recent years, it has been revealed that astrocytes perform a significantly wider range of functions than previously appreciated. Interest in astrocyte function has increased dramatically because of their newly discovered roles in synapse formation, maturation, efficacy, and plasticity. Today, astrocytes are recognized as multifunctional cells with well-defined essential neuron supporting functions. Mounting evidence suggests that these versatile cells participate in a multitude of diverse processes in the central nervous system (CNS). These roles include regulating blood flow, providing much needed energy to neurons, and supplying the building blocks of neurotransmitters that fuel synapse activity [1]. However, the roles of astrocytes are not restricted to supporting neuronal function [2]. The addition of their role in synaptic function to the known repertoire of astrocyte activities over the past decade has enhanced our conception of their seminal importance in normal functioning of the adult brain. More comprehensive reviews highlighting astrocyte function include Jacobs et al. [3], Wang and Bordey [4], and Kimelberg [5].

In the developing nervous system, the assembly of synaptic circuits is a complex and dynamic process, requiring the coordinated exchange of signals between pre- and postsynaptic neurons and neighbouring glia [6]. The formation, maintenance, and modulation of synaptic connections are required for normal CNS function and ongoing plasticity. In the diseased nervous system, however, the structural and functional integrity of synaptic connections is often modified or lost, resulting in profound cognitive and behavioral deficits. Yet until recently, no exact roles had been identified for astrocytes in the pathogenesis of specific CNS diseases. While some aspects of the mechanisms underlying the formation, maintenance, and plasticity of CNS synapses in the developing and diseased nervous system have been elucidated, many more remain enigmatic. 

As our knowledge about astrocytic function in normal physiology has expanded, exploration into their likely role in disease pathology has followed. In the case of fragile X syndrome (FXS), a compelling case can be made for the abnormal dysfunction of astrocytes. FXS is the most common form of inherited mental impairment, and it typically results from the transcriptional silencing of the *FMR1* (*fragile X mental retardation 1*) gene and loss of the encoded protein, FMRP (fragile X mental retardation protein) [7]. FXS symptoms include neurodevelopmental delay, anxiety, hyperactivity, and autistic-like behavior. FMRP was once thought to be expressed solely in neurons; however, it was later shown to have specific roles in astrocytes. In fact, it appears that the expression of FMRP is developmentally regulated. Pacey and Doering [8] found that FMRP is expressed in early development in cells of the glial lineage both *in vitro* and *in vivo*.

Although few studies focus specifically on the role of astrocytes, recent work provides important examples of how a better understanding of astrocyte biology during development can enhance our knowledge about human disease. In this paper, we discuss the landmark findings and recent advances in our understanding of astrocytes and their featured roles in regulating synapse formation, maturation, and synaptic transmission. Further, we assess how astrocytes contribute to the extensive plasticity that occurs during development, highlighting the dynamic morphology of astrocyte processes and their involvement in synaptic development. Lastly, we explore the means by which perturbations in astrocyte function may contribute to neurological diseases, such as FXS, in the context of synaptic defects. We propose here that, by investigating the precise roles of astrocytes during neurological disease, we are likely to achieve a broader understanding of how the brain works, in addition to new insights into disease prognosis, diagnosis, and treatment.

## 2. Astrocyte Diversity

Astrocytes, or astroglia, are named with the Greek root word “astro,” which means star. They were so named due to their “stars in the night sky” appearance obtained from Golgi stained samples [9]. In the late nineteenth century and the early twentieth century, Camillo Golgi and Santiago Ramón y Cajal noticed that, although different astrocytes share a stellate feature, their morphology is extremely diverse, perhaps as diverse as neurons. Since Cajal's time, modern scientists have confirmed the morphological diversity of astrocytes *in vitro* and *in vivo* [10, 11].

Astrocytes are divided into two main classes distinguished on the basis of their morphology and primary location [12, 13]. Protoplasmic astrocytes are classically found in the grey matter of the brain. Their processes, which are long, thick, and highly ramified, are closely associated with synapses as well as blood vessels [11]. In the hippocampus, protoplasmic astrocytes ensheath more than half of the synapses, most of which are excitatory [14]. The other subtype is composed of fibrous astrocytes found mainly in the white matter of the brain, where their processes pass between nerve fibers. In contrast to protoplasmic astrocytes, fibrous astrocyte processes are long, cylindrical, smooth, and branch infrequently.

Astrocytes are also far more morphologically complex than initially appreciated [15]. The morphology of a mature mammalian astrocyte is spectacular. From the cell soma radiate primary branches that gradually divide into finer and finer processes to generate a dense network of delicate terminal processes, which associate very closely with synapses. A number of immunological markers have been used over the years to characterize astrocyte morphology. Until recently, our understanding has been predominantly based on classical immunostaining with the widely used astrocyte marker GFAP (glial fibrillary acidic protein, an intermediate filament protein), which grossly underestimates the complexity of astrocytes and their interactions with neurons and other cells [16]. GFAP only reveals the structure of primary branches, which represent a meager ~15% of the total volume of the astrocyte. Other markers include ALDH1Ll (aldehyde dehydrogenase 1 family, member L1) [17], Glt-1 (glial glutamate transporter 1), and GLAST (glutamate-aspartate transporter) [18]. To date, no marker has been identified that is expressed exclusively in mature astrocytes. Moreover, no pan-astrocytic marker has been identified with which to determine the fraction of astrocytes that are GFAP+, although recent studies on ALDH1L1 seem promising [17].

Recent physiological and gene expression profiling studies indicate that astrocytes, like neurons, are a diverse cell population with distinct properties in different brain regions and at different periods of development [19]. For instance, astrocytes are crucial at every step of neural development. They function as neural stem cells and guide axon projections; they promote synapse formation and maintain neuronal survival [20, 21]. Astrocytes also differ in their proliferation potential. Subsets of astrocytes, or astrocyte-like cells, in the adult subventricular zone (SVZ) and in the subgranular zone (SGZ) of the dentate gyrus of the hippocampus act as neural stem cells, whereas most astrocytes in other parts of the adult brain do not normally proliferate [22]. Heterogeneity of astrocytes, however, is not exclusive across brain regions, as it can also exist within the same areas of the brain [23]. The number and size of astrocytes in the brain also vary between species relative to species brain size and cognitive ability. For example, the human brain contains several more populations of astrocytes than the rodent brain, and human astrocytes are threefold larger than their rodent counterparts [24]. Therefore, these classifications may not be adequate to appreciate the full extent of astrocyte diversity.

Astrocytes have unique cytoarchitectural and phenotypic features that ideally position them to sense their surroundings and respond in dynamic ways to changes in their microenvironment [25]. Astrocytes are, therefore, well suited to share synaptic function with neurons as they extend numerous processes, forming highly organized anatomical domains with little overlap between adjacent cells. They are also interconnected into functional networks via gap junctions. The territory of a single astrocyte is estimated to contact between 300 to 600 dendrites and upwards of 10^5^ synapses [16, 26]. This extensive synaptic interaction not only ensures that astrocytes are able to fulfill their metabolic support roles but also positions astrocytes to directly influence the structure and function of the synapse [27]. While some astrocyte processes (which express a wide range of receptors and ion channels) closely ensheath synapses, others are in close contact with intraparenchymal blood vessels via specialized processes called endfeet. In line with this, astrocytes have been shown to play an important role in neurovascular and neurometabolic coupling [23].

## 3. Astrocytes Influence Synapse Formation**** and Development

In the past decade, astrocytes have emerged as essential regulators of synaptic connectivity. The formation of synaptic contacts is paramount for the proper development and function of the CNS. Although most neurons are produced during embryonic stages, the major waves of synaptogenesis follow and depend on astrocyte production. Given their proximity to synapses, astrocytes can directly promote and regulate these processes through both secreted and contact-mediated signals.

### 3.1. Secreted Signals

The traditional assumption that neurons are intrinsically able to form synapses led early studies on synaptic development to focus on neuronal signals and surface molecules. Remarkably, neurons cultured with media conditioned by astrocytes control the number and effectiveness of synapses [28–30], indicating that soluble factors secreted from astrocytes play an important role in synapse formation. Some of the factors released by astrocytes that mediate these effects have been identified. These include matricellular proteins [31], such as thrombospondins (TSPs-1–4), SPARC, SPARC-like 1 (Hevin), and tenascin C, which are all expressed by astrocytes in the CNS of rodents.

A possible role for glial involvement in CNS synaptogenesis was first elucidated by a series of studies on rat retinal ganglion cells (RGCs). Cholesterol complexed to apolipoprotein E (ApoE) released by astrocytes increases the number of glutamatergic synapses in RGC cultures. When cholesterol is applied directly to cultured RGCs, the frequency of spontaneous synaptic events increases. The researchers further demonstrated that cholesterol acts to increase the quantal content of synaptic vesicles and the overall efficacy of vesicle release [32]. This is in concert with other findings that cholesterol is an essential component of synaptic vesicle production whose presence serves as a limiting factor in vesicle formation [33].

The RGC culture technique has also been used to identify other key synaptogenic-secreted factors including Thrombospondins 1 and 2 (TSP-1 and TSP-2), members of oligomeric extracellular proteins. Christopherson and colleagues identified TSPs as the signals coming from astrocytes that can induce an increase in synapse number [34]. When directly applied to RGC cultures, TSP-1/2 increased the number of immunochemically identified synapses nearly 3-fold. Immunodepletion of TSPs from astrocyte-conditioned medium (ACM) decreased its synaptogenic effect down to control levels indicating that TSPs are the key synaptogenic component of ACM. 

The expression of TSP-1/2, which is elevated in the developing brain when the majority of synapses are formed (during postnatal week 1), ceases in the mature adult brain (by postnatal week 3). This suggests that astrocytes downregulate pathways that strongly promote synapse formation when the synaptogenic period of neurons is reduced, and other TSP genes may be functioning to stabilize synaptic structures. Besides TSPs 1 and 2, other TSPs (TSPs-3–5) are detected in mammals [35]. Astrocytes have been found to express mRNAs for TSP-3 and -4. In contrast to other TSP-s, TSP-4 expression is only detected in mature astrocytes after P17 [17]. This suggests that TSP-4 could represent the adult isoform of TSP in the CNS and is important for the control of synaptogenesis and enhanced plasticity in the adult brain. Recently, gabapentin receptor *α*2*δ*-1 has been identified as the TSP receptor responsible for mediating excitatory CNS synaptogenesis [36]. Despite the critical role of TSPs in promoting synaptogenesis, additional signals are likely required for synapse maturation, as TSP-induced synapses are ultrastructurally normal, but postsynaptically silent, underscoring the complexity of astrocyte contribution to synapse formation. A more recent study has identified two closely homologous glypicans, glypican-4 and glypican-6, as astrocyte-secreted proteins that are sufficient to increase AMPA glutamate receptor levels on synapses, thus inducing postsynaptic function [37].

Additionally, tenascin-C (TN-C), another extracellular matrix glycoprotein, seems to play a role in synaptogenesis and synaptic function [38, 39]. TN-C is highly expressed by astrocytes during early stages of development, while its expression ceases in the adult CNS [40], with the exception of specific cell populations, particularly those in close proximity to areas of active neurogenesis, such as the hippocampus, subventricular zone borders, and the rostral migratory stream. Following stimulation of synaptic activity, TN-C was found upregulated in the hippocampus within a few hours [41]. In TN-C knockout mice, stimulation of Schaffer collaterals resulted in a reduction of long-term potentiation (LTP) at CA1 synapses, whereas CA1 long-term depression (LTD) was completely abolished [42, 43]. These expression patterns reveal important roles for TN-C in the remodeling of the CNS, both during development and in adulthood.

Notably, astrocyte-secreted factors do not act exclusively to promote excitatory synaptogenesis. In fact, recent studies reveal astrocyte contributions to inhibitory synapse formation and function in cultured hippocampal neurons. While astrocyte-expressed extracellular matrix protein Hevin has been found to induce the formation of synapses in cultured RGCs [44, 45], its homolog, SPARC, which is also secreted by astrocytes, antagonizes the synaptogenic function of Hevin, thereby acting as a negative regulator of synapse formation [44]. SPARC expression is typically high in early development, where it then becomes downregulated in certain parts of the brain by the time of synaptogenesis. Alternatively, Hevin expression increases with development in agreement with synapse formation and is also present in adulthood, most likely functioning in the maintenance of existing synapses. Unlike TSP-1 and TSP-2, the expression of which is decreased during maturation, Hevin and SPARC mRNA levels remain high even in the adult. Taken together, the secretion of both positive and negative regulators of synapse formation allows astrocytes to regulate the timing and location of synapse formation with greater precision.

Moreover, a recent study has provided evidence that astrocytes play a role in the elimination of redundant synapses during development. In the developing postnatal brain and retina, immature astrocytes seem to be a source of a signal that triggers the expression of complement component C1q in developing neurons [46]. C1q's best-known role in the innate immune system is to opsonize or “tag” unwanted cells or debris for elimination. C1q localizes to synapses that are thus tagged for elimination through the activation of the complement cascade and deposition of C3b, an opsonin derived from the proteolytic activation of the complement component C3. Mice deficient in C1q or the downstream complement cascade protein C3 exhibit large sustained defects in CNS synapse elimination, as shown by the failure of anatomical refinement of retinogeniculate connections and the retention of excess retinal innervation by lateral geniculate neurons. Also, C1q-deficient mice show enhanced neocortical excitatory synaptic connectivity and epileptiform activity [47]. Together, these findings implicate a role for astrocytes during the critical period when neural circuits are formed.

### 3.2. Contact-Mediated Effects

While astrocyte-secreted factors induce the formation and function of synapses, other evidence proposes further regulatory roles for astrocytes through contact-mediated mechanisms. An elegant study by Hama et al. [48] provided evidence that astrocytes upregulate synapse formation by the process of adhesion. Local contact with astrocytes via integrin receptors facilitated excitatory synaptogenesis through the activation of protein kinase C (PKC) in individual dissociated hippocampal neurons. The researchers observed that PKC activation, while initially focal, subsequently spread throughout the entire neuron. Thus, propagation of PKC signaling could signify an underlying mechanism for global neuronal maturation following local astrocyte adhesion.

Astrocyte processes, which are highly mobile, contribute to the stabilization of new synapses during synaptogenesis. Astrocytes may induce local structural and functional modifications of dendritic segments or individual synapses through a contact-mediated mechanism involving bidirectional ephrin/EphA signaling [49–51]. Membrane-bound ligands on astrocytes, such as ephrin-A3, have been shown to upregulate spine morphology in the hippocampus, suggesting local activation of EphA receptors on spines by astrocytic ephrin-A3. Dendritic spines are small protrusions visible on dendrites of neurons that serve as postsynaptic sites for excitatory input [52–54]. Live imaging of organotypical hippocampal slice preparations showed that astrocytes rapidly extend and retract fine processes to engage and disengage from postsynaptic dendritic spines [55]. Studies with two-photon microscopy that tracks the dynamics of astrocyte processes and the fate of dendritic protrusions also revealed contributions of astrocyte contact [56]. Dendritic protrusions with astrocyte contacts had a longer lifetime and were morphologically more mature. Thus, dendritic protrusive activity and transient contacts with astrocytes act to stabilize newborn synapses and promote subsequent spine maturation. Spine dynamics are largely controlled through changes in cytoskeletal proteins [57]. Expressing a dominant negative mutant Rac1, a GTPase that mediates actin motility, reduces astrocyte process motility and provides evidence that cytoskeletal rearrangements underlie motility, similar to mechanisms of spine extension and retraction [56, 57].

The development of inhibitory synapses can also be modulated by astrocyte contact. Liu et al. [58] showed that local contact between neurons and astrocytes significantly increased the amplitude and density of GABA_A_ currents in developing hippocampal neurons. This contact-dependent increase in GABAergic synaptic activity relied on Ca^2+^ signaling in astrocytes. In addition, astrocytes were shown to regulate Cl^−^ gradient in cultured spinal cord neurons and convert GABAergic synapses from excitatory to inhibitory [59]. This finding is particularly exciting given the importance of local GABAergic inhibitory circuits in both activity-dependent wiring of developing neural circuits and the consolidation of critical period plasticity [60, 61].

Overall, these studies reveal that contact-mediated signaling between astrocytes and neurons is important for the structure and maintenance of synaptic connections and suggests a model in which physical and molecular interactions between neurons and astrocytes provide instructive cues that control synapse formation, morphology, and plasticity.

## 4. Astrocytes Modulate Synaptic Transmission

As our understanding of the extent of their influence at the synapse unfolds, it is much more apparent that astrocytes are well poised to modulate multiple aspects of synaptic plasticity than was previously imagined. A turning point in our understanding of astrocytes was elicited by the recognition of their active communicative properties [62–64]. Networks of astrocytes can act in concert to influence transmission among neighbouring synapses. Astrocytes, which are bidirectional, can communicate and exchange information with both pre- and postsynaptic elements. Communication is primarily controlled by the change in Ca^2+^ concentrations, causing excitability within the astrocyte [64–66].

Astrocytes use their ability to respond to neurotransmitters and secrete neuromodulators to actively regulate a number of processes involving synaptic plasticity [67–69]. In addition to secreting factors that influence and modulate synapse formation, astrocytes are known to release factors that can directly affect synaptic transmission. Briefly, of the gliotransmitters released by astrocytes [70], the most well characterized and extensively reviewed are glutamate [71, 72], adenosine triphosphate (ATP) [73], and D-serine [74, 75]. Glutamate serves as the principal excitatory neurotransmitter in most regions of the CNS, and its release from astrocytes has been shown to modulate synaptic transmission [76]. Glutamate released from neurons activates metabotropic glutamate receptors on astrocytes, leading to an increase in astrocyte Ca^2+^ concentrations and a subsequent astrocytic release of glutamate. D-serine, perhaps the most interesting, is an important neurotransmitter that serves as a coagonist with glutamate, promoting NMDA (N-methyl-D-aspartate) receptor activity at synapses in the hypothalamus [75]. Moreover, astrocytes release ATP to communicate with each other and other glia by activating purine receptors localized on neighbouring cells [73]. These findings have led to the establishment of a new concept in synaptic physiology, the tripartite synapse, in which astrocytes exchange information with neuronal synaptic elements [6, 67, 77]. Consequently, astrocytes are an integral part of the synapse, being involved not only in passive homeostatic control of adequate conditions for synaptic function, but also actively in synaptic function [78].

## 5. Astrocytes and Pathology: Contributions**** to Neurological Disorders

With an evident role of astrocytes in normal neural function at all cellular and molecular levels, it is not surprising that astrocytes contribute in some capacity to almost all pathological conditions of the nervous system [79–84]. For most disorders, it remains unclear whether astroglial changes are causative of the disease or if they merely represent an accompanying phenomenon. Accordingly, astrocyte-dysregulated function has been fundamentally linked with the progressive pathology of ischemic stroke, epilepsy, and to a number of neurodegenerative disorders including, but not limited to, amyotrophic lateral sclerosis, Huntington's disease, and Parkinson's disease. Further involvement of astrocytes has also been implicated in the development of neurodevelopmental disorders such as Rett syndrome (RTT), Down syndrome (DS), Fragile X (FXS), and autism. Among these conditions, FXS has emerged as the prototypical disorder in which to study how altered signaling may lead to synaptic defects and dysfunctional neural circuitry underlying pathology [85]. Both dysregulated astrocyte signaling and abnormal synaptic function stand as prominent contributing factors to the learning disability phenotype expressed in FXS.

## 6. Fragile X Neurobiology

Fragile X syndrome (FXS) is the most common form of inherited intellectual disability [7]. It affects approximately 1 in 4,000 males and 1 in 6,000 females and is characterized by cognitive impairments, attention deficits, and autistic-like behaviors [86]. FXS is caused by an expanded CGG trinucleotide repeat in the 5′ untranslated region of the *FMR1* gene leading to gene silencing and the consequent loss of FMRP expression [87, 88].

**Figure 1:**
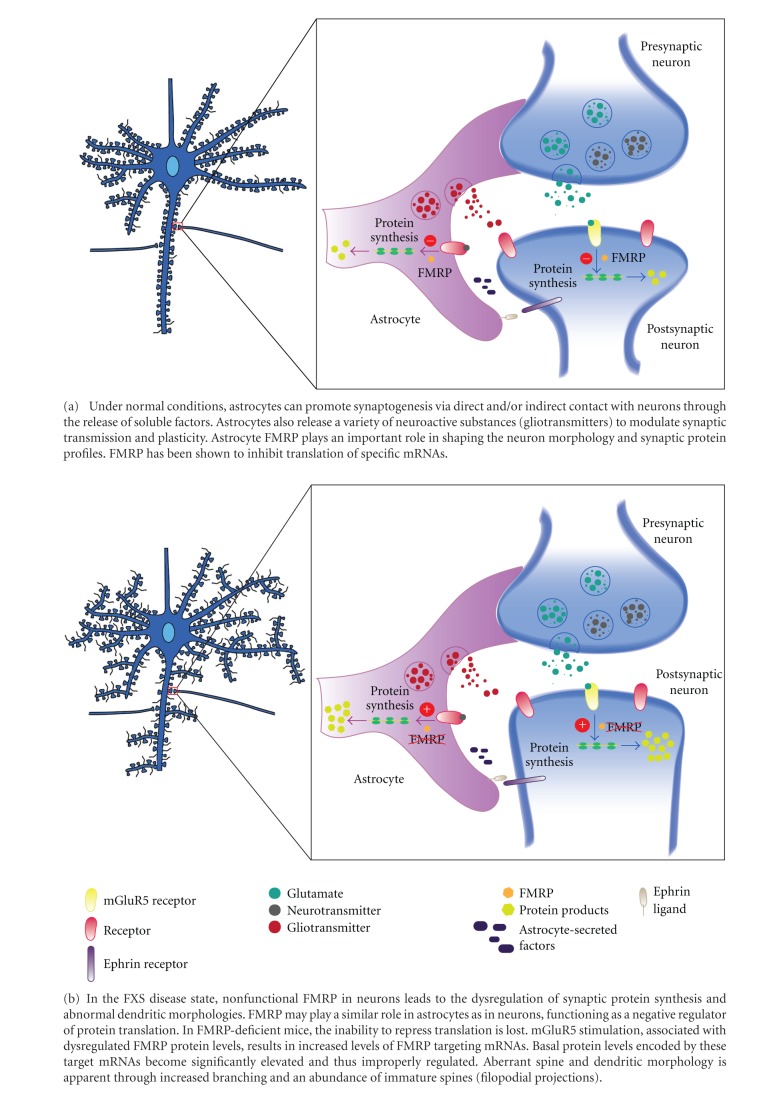
The role of astrocytes in FXS. It is becoming increasingly apparent that, in addition to presynaptic terminals and postsynaptic dendritic spines, synapses contain a third element: the fine processes of the astrocyte, which intimately enwrap the first two elements.

To understand the etiology of the synaptic phenotypes that accompany FXS, it is first important to discuss the purported function of FMRP. FMRP acts as a regulator for the transport and local translation of specific synaptic mRNAs in response to neural stimulation [89]. FMRP is found in growth cones, immature axons, and mature dendrites, as well as dendritic spines [90]. Accumulating evidence suggests roles for FMRP in synapse development, elimination, and plasticity. The loss of FMRP results in the aberrant expression of its mRNA targets, which in turn leads to functional deficits that characterize FXS. The reason that FMRP has been implicated in synaptic plasticity is on the basis of dendritic spine abnormalities and exaggerated long-term depression (LTD) displayed by *FMR1 *mutant mice. This finding led to the “mGluR” theory of FMRP, whereby synaptic signaling of metabotropic glutamate receptor 5 (mGluR5) leads to the localized translation of *FMR1* mRNA [91]. As such, the newly synthesized FMRP acts as a translational repressor of specific target mRNAs, resulting in the downregulation of mGluR5 activity through a negative feedback loop [92] (Figure 1). Several exceptional reviews on the genetic and clinical features of FXS or molecular functions of FMRP include Bear [93], Huber et al. [92], Garber et al. [94], and Bassell and Warren [89].

### 6.1. FXS Animal Models

Current knowledge surrounding the pathophysiology of FXS has been greatly advanced by the development of animal models [95]. These transgenic animals do not carry the trinucleotide expansion but do have functional deletions of FMRP. The first model developed was the *FMR1* knockout (KO) mouse [96], which recapitulates behavioural and cognitive deficits reminiscent of the human condition. Drosophila and zebrafish models also exist and have contributed to our understanding of the conserved roles of FMRP in neural development [97–99]. Although they are not perfect models of the human disease, they have helped to reveal the cellular and molecular mechanisms underlying FXS, and they have immensely enhanced glial-neuronal research.

### 6.2. FXS Spine Dysgenesis

During development, the first postnatal weeks of the mammalian brain are characterized by extensive plasticity. Selective elimination or pruning of inappropriate synaptic connections occurs for the proper formation and establishment of neural circuitry. Current models regarding the neurobiological changes that underlie Fragile X have largely focused around the synapse. This is based in part on the structural synaptic changes and alterations in synaptic function, which are observed in human patients and FXS animal models.

Filopodial spine morphology has long been a common hallmark of disease. Spines develop around the time of synaptogenesis and are dynamic structures that continue to undergo remodeling over time. Developmental changes in the shape of dendritic protrusions reflect the progressive replacement of thin, elongated, and highly motile filopodia, characteristic of immature neurons, with more stable spines that acquire a mature morphology [100]. Spine morphogenesis is fundamental to the development of neuronal networks and the regulation of synaptic plasticity.

Some of the first neuroanatomical findings associated with mental impairment were alterations in dendritic spine structure [101]. The first such evidence of altered synapse structure in FXS came from analysis of postmortem cortical tissue, which exhibited an increased number of dendritic spines relative to control individuals [102]. This data revealed that excitatory synapse number was increased in FXS patients and further provided a potential mechanism for the increased rates of epilepsy in FXS. It was additionally noted that a large proportion of the spines of FXS patients appeared abnormally long, thin, and tortuous, a phenotype reminiscent of the immature spine precursors (filopodia), and indicative of alterations in synapse development and/or function. At this point, it was not clear if the excess filopodia-like spines in FXS represented functional synapses or immature synapse precursors. 

Much of the evidence for a role for FMRP in synaptic and neurite pruning is derived from the *Drosophila melanogaster* model of FXS (dFXR). During development, FMRP has been shown to control the pruning of immature dendrites in developing neurons. In support of a pruning function for dFXR, most neurons of dFXR null flies exhibit an overgrowth and elaboration of axons and dendrites into the peripheral and CNS [98, 103–106].

Parallel to human studies, work with the* FMR1* KO mouse has largely confirmed the spine phenotype observed in FXS patients. Numerous studies agree that *FMR1* mutant brains display an increase in long, thin, immature dendritic spines [102, 107] mirroring human neuroanatomical abnormalities [102]. It is important to note that many of these defects in spines and in synaptic/circuit plasticity occur during critical periods of development in the first postnatal weeks, coinciding with the maximal expression of FMRP. However, the existence and/or magnitude of the spine alterations in the *FMR1 *KO mouse varies according to brain region, developmental age, and genetic background, indicating the complex and multifactorial regulation of spines.

In a study by Cruz-Martin et al. [108], spines of L2/3 layered pyramidal neurons were imaged at various developmental stages, and it was revealed that *FMR1* KO mice demonstrated a delay in the stabilization of dendritic spines, due to high turnover during the second postnatal week [108]. This happens to correspond to the time when FMRP protein expression is highest in the cortex [109]. In the absence of FMRP, hippocampal neurons have fewer spines that colocalize with synaptic markers, which suggests a loss of functional spines [90]. This provides compelling evidence that FXS might be caused by a failure in the transition from filopodia (earliest dendritic protrusions) to mature spines, consequently resulting in an increase of immature synapses. The failure of spines to stabilize during the critical period in the barrel cortex strongly suggests that* FMR1* KO mice could have problems in maintaining the proper balance between stable and dynamic connections that is necessary to establish mature synapses. Since dendritic spines are the primary sites of excitatory synapses and information exchange in the CNS, perturbations in their structure and function can result in synaptic and circuit alterations leading to disrupted brain function and pathology.

## 7. Astrocyte Involvement in Fragile X

While it has been recognized that astrocytes play multiple critical roles in the regulation of normal CNS function, the possibility that astrocyte-specific dysfunction might cause diseases that manifest as pathologies of neurons is a relatively recent idea. Previously, it was thought that FMRP expression in the brain was exclusively confined to neurons. FMRP had been reported in oligodendrocyte precursor cells, but not mature oligodendrocytes [110]. Our laboratory initially identified FMRP in the astrocyte lineage in the FXS mouse [111]. When studying stem and progenitor cells from the brains of wild-type (WT) and knockout (KO) FXS mice, approximately half of the cells in culture coexpressed FMRP and GFAP. Parallel immunocytochemical studies *in vivo* also showed the coexpression of FMRP and GFAP in the embryonic and adult developing hippocampus.

**Figure 2:**
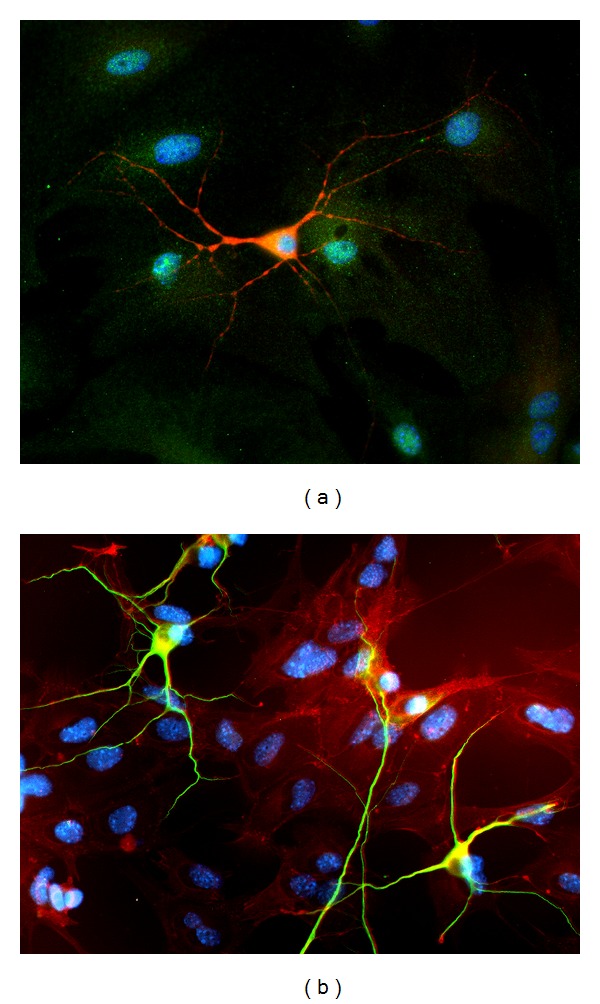
Examples of Fragile X astrocyte-neuron cocultures. (a) *FMR1* WT astrocytes + WT neurons double labeled with MAP-2 (neuron in red) and gephyrin (astrocytes in green); (b) *FMR1 *KO astrocytes + WT neurons identified with MAP-2 (neurons in green) and GFAP (astrocytes in red); DAPI—nuclei (blue).

With the identification of FMRP in astrocytes and knowledge of their role in synaptogenesis, our laboratory was prompted with further experiments to explore neuronal development and synapse formation in FXS [112]. Utilizing a coculture design adapted by Jacobs and Doering [113], hippocampal neurons (E17) and cortical astrocytes (P0-1) were independently isolated to explore four different combinations of neuronal-astrocyte cultures (WT neurons + WT astrocytes, WT neurons + *FMR1* KO astrocytes, *FMR1* KO neurons + WT astrocytes, *FMR1 *KO neurons + *FMR1* KO astrocytes). The cells were grown for 7, 14, or 21 days *in vitro* and then processed for immunocytochemistry to analyze morphological and synaptic profiles. Examples of the cocultures are shown in Figure 2. These experiments are novel and exciting as they are the first to establish a potential role for astrocytes in the altered neurobiology of FXS.

The first group of experiments focused on neurons in each of the four combinations to elucidate the effects of FMRP on dendritic morphology and excitatory synapse expression. The neurons were studied with antibodies directed against the neuronal (dendritic) marker, MAP-2, the presynaptic protein synaptophysin, and excitatory postsynaptic protein, PSD-95, respectively. Through Sholl analyses, morphological assessments were performed on neurons under parameters of dendritic branching and the area of the cell body. Synaptic protein distribution was determined by the quantification of synaptic puncta (spots of intense staining). WT neurons grown on *FMR1 *KO astrocytes exhibited significantly altered dendritic arbor morphologies, with a shift toward a more compact and highly branched dendritic tree. Specifically, WT neurons grown on *FMR1 *KO astrocytes resulted in a decrease in the length of the longest primary dendrite and area covered by dendritic arbor, and an overall increase in branch number and density in comparison to their WT counterparts. These neurons also displayed a significant reduction in the number of pre- and postsynaptic protein aggregates. However, when the *FMR1* KO neurons were cultured with WT astrocytes, the alterations in dendritic morphology and synaptic protein expression were remarkably prevented. In fact, their morphological characteristics and synaptic protein expression approached the appearance of normal neurons grown with WT astrocytes. These experiments were the first to suggest that astrocytes contribute to the abnormal dendritic morphology and the dysregulated synapse development seen in FXS.

In the next phase of this research, we wanted to determine if these altered characteristics represented a developmental delay imparted by the *FMR1 *KO astrocytes [114]. Focusing on WT neurons grown in the presence of WT or *FMR1* KO astrocytes, we evaluated the dendritic arbor morphology and synaptic protein expression at 7, 14, and 21 days in culture. Our results revealed that WT neurons grown with *FMR1* KO astrocytes displayed significantly altered morphological and synaptic protein profiles at 7 days (when compared to the WT condition). Strikingly, by 21 days in culture, these differences were no longer significantly different from normal. In light of these findings, it appears that astrocytes in the FXS mouse may contribute to the altered characteristics of neurons seen in FXS in a developmentally regulated manner. Thus, these results suggest that timing is crucial in brain development. Despite these outcomes, it is noteworthy that conclusions about synapse maturity cannot be drawn. It is possible that the increase in synapses observed in the neurons grown on *FMR1* KO astrocytes reflects an increased number of immature synapses. Given that the dendritic spine is the site for the majority of excitatory synapses, this finding would be in agreement with numerous studies that identified neurons in FXS with an abnormally high number of immature dendritic spines. As a note, the methods used in the current study did not permit the assessment of alterations in dendritic spine morphology.

### 7.1. Outstanding Questions and Future Approaches

Understanding the role of astrocytes in human neurological diseases requires a comprehensive picture of how astrocytes develop and what roles they play in development. Given these findings, it is highly plausible that FXS astrocytes lack functional FMRP, specifically at a time during development when astrocyte support of neuron growth and synapse formation is vital, and that this lack of FMRP could contribute to the abnormal neuron phenotype seen in FXS. However, it is uncertain whether the alterations in astrocytes are due to a lack of FMRP or if they are abnormal because they develop and function in a diseased microenvironment. Also, if the absence of FMRP in astrocytes is the primary source of dysfunction, how are these effects translated to neurons? For instance, is astrocyte-neuron signaling disrupted due to a lack of astrocyte FMRP? How, where, and when do these signals act? Is the abnormal astrocyte-neuron communication mitigated by a membrane associated or a soluble factor? Could it be a combination of both direct and indirect contact? These questions, among many others, about the FXS astrocyte are now important targets for FXS research. The answers will allow us to gain a full understanding of the underlying neurobiology that contributes to the morphological phenotype seen in FXS and in the potential of a future treatment for individuals with FXS.

Recent evidence indicates that the interface between astrocytes and neurons is necessary for normal synapse development, including synaptic pruning. Dendritic spines, which are highly dynamic during development, become more stable in the adult brain; thus, a correlation exists between age-dependent spine dynamics and the plasticity of the brain. This decrease in spine motility in the mature brain could be attributed to the close association of astrocytes with synapses, with astrocytes providing both physical constraints that inhibit spine movement as well as molecular interactions that stabilize spines. Importantly, EphA4R (expressed on dendritic spines) interacts downstream with members of the Rho/Ras pathways, suggesting that EphAR/ephrin-A interactions may underlie aspects of actin-driven astrocyte motility observed during synapse formation [27, 115]. Interferences in these interactions may result in the destabilization of newly formed spines [49]. Therefore, this raises the possibility that* in vivo *defects in dendritic spine development are at least partly related to neuron-glia interactions during development.

Astrocyte involvement has also been fundamentally implicated in neurodevelopmental disorders such as RTT and DS. A common finding in many of these studies is that astrocyte dysfunction has profound non-cell-autonomous effects on surrounding neurons. In fact, synaptic function and structure may be a converging point of malfunction. RTT, which is an X-linked neurodevelopmental disorder, is caused by the loss of the transcriptional repressor methyl-CpG-binding protein (MeCP2). A study by Ballas et al. [116] showed that wild-type hippocampal neurons cocultured with cortical astrocytes or conditioned medium from *Mecp2*-deficient mice had abnormally stunted dendrites, suggesting that *Mecp2*-deficient astrocytes may dominantly affect normal neuronal development. Furthermore, in DS patients, cognitive deficits have been associated with structural changes in the architecture and alterations in dendritic spine number. Garcia et al. [117] found that DS astrocytes are directly involved in the development of spine malformations and reduced synaptic density. These researchers also indicated that the astrocyte-secreted protein TSP-1 possesses a potent modulatory effect on spine number and morphology. Taken together, these studies serve to identify astrocyte dysfunction as a significant factor of spine and synaptic pathology.

Future experiments will focus on the assessment of dendritic spines in FXS and the role of direct/indirect neuronal-astrocyte cell contact in the altered developmental sequences that we observed in our tissue culture paradigm. It is highly conceivable that the absence of astrocyte FMRP would directly affect spine morphology or dynamics via dysregulated protein synthesis, and a defect in the maturation of dendritic spines could explain deficits in the intellectual ability seen in individuals with FXS.

## 8. Closing Remarks

Armed with novel experimental techniques, powerful imaging tools, and a better understanding of astroglia, neuroscientists are uncovering a new view of the synapse. Neuroscientists are now in a better position to explore and uncover the long-standing mysteries of astrocytes and gain new insights into the cellular and molecular underpinning of the nervous system. The recent findings discussed in this paper place astrocytes in an important position to actively exchange signals with neurons and other glial cells to coordinate synaptic networks. Astrocytes secrete soluble factors that enhance synaptogenesis and release neuroactive molecules that mediate plasticity. Both astrocyte contact and secreted factors are important in regulating synapse formation and function. While studies help to distinguish the effects of astrocyte contact from secreted factors on neuronal form/function, it is unlikely that they are separate *in vivo*. Also, given the central role of the synapse in neuronal communication and plasticity, it comes as no surprise that dysregulation of the synapse accounts for many, if not most, of pathological and developmental disorders in the brain. Thus, the involvement of astrocytes and how they interface with neuronal circuitry should be taken into consideration when interpreting future studies in the pathophysiology of FXS and/or other related neurological diseases.

A unifying theme from these recent findings is that astrocytes can promote the development and plasticity of synaptic circuits. Much of the current literature surrounding FXS focuses on synaptic control of protein synthesis because it appears to be proximal to the biology of FMRP and the pathogenesis of the disease in multiple animal models. In addition to targeting synaptic protein synthesis, other therapeutic approaches show promise, for example, in changing the balance of excitation to inhibition by enhancing GABA signaling [118]. Whether different approaches will converge on the same pathophysiological processes or whether they will target distinct aspects of the disease remains to be determined. As we continue to expand our understanding, insights into how these mechanisms may be perturbed in FXS and other diseases states may pave the way for promising future therapeutic interventions and treatments. Potential modes of pharmacological therapy should indeed concentrate on the astrocyte as a “gatekeeper” of neuronal health and function.
